# Morphosemantic activation of opaque Chinese words in sentence comprehension

**DOI:** 10.1371/journal.pone.0236697

**Published:** 2020-08-12

**Authors:** Jian Huang, Yiu-Kei Tsang, Wanting Xiao, Suiping Wang

**Affiliations:** 1 School of Psychology, South China Normal University, Guangzhou, China; 2 Key Laboratory of Brain, Cognition and Education Sciences (South China Normal University), Ministry of Education, Guangzhou, China; 3 Center for Studies of Psychological Application, South China Normal University, Guangzhou, China; 4 Guangdong Key Laboratory of Mental Health and Cognitive Science, South China Normal University, Guangzhou, China; 5 Department of Education Studies, Hong Kong Baptist University, Hong Kong, China; University of Windsor, CANADA

## Abstract

Two cross-modal priming experiments were conducted to investigate morphological processing in Chinese spoken word recognition during sentence comprehension. Participants heard sentences that contained opaque prime words and performed lexical decisions on visual targets that were related to second morpheme meanings of opaque words or whole-word meanings. The targets were presented at the auditory onset of the second morphemes or the subsequent syllables after the opaque primes to examine the time course of effects. In a neutral sentence context (Experiment 1), opaque word morpheme meanings produced morphological priming on target word recognition, which preceded lexical priming. When context biased toward whole opaque words (Experiment 2), morphological priming disappeared, while the effect of lexical meanings remained significant and emerged earlier than the effect of lexical meanings in the neutral context. These findings suggest that morphemes play a role in Chinese spoken word recognition, but their effects depend on the prior context during sentence comprehension.

## Introduction

Chinese is a morphologically simple language. Inflection and derivation are rare. For example, there are no gender markers, and word class is mostly determined by context rather than suffixation. In contrast, compound words are more common. Over 60% of all Chinese words are bimorphemic [1, 2]. Chinese is also widely referred to as a morphosyllabic language because there is usually a simple correspondence among characters, syllables, and morphemes. For example, the character “手” is pronounced as the monosyllable/shou3/(in Mandarin Chinese) and functions as a morpheme carrying the “hand” meaning in compound words such as “手指-finger” and “手枪-handgun”. Two-character (disyllabic) morphemes are rare exceptions (e.g., “垃圾”/la1ji1/is the “rubbish” morpheme in “垃圾桶-rubbish bin”). Overall, morpheme boundaries are highly salient in Chinese [3]. They are marked by physical space in written text and by syllable onset in speech. These features have led to the proposal that multimorphemic words in Chinese are recognized through morphological decomposition [4, 5].

On the other hand, Packard [6] argued that morphological decomposition is highly inefficient in Chinese. Specifically, he reasoned that while each character and syllable is mapped to a morpheme in most cases, the mapping may not be one-to-one. It has been estimated that over 50% of characters have multiple meanings (i.e., these characters are homographic morphemes; [2, 7]). For example, the character “教”/jiao4/is the “teach” morpheme in “教室-classroom” and “教练-coach” but the “religion” morpheme in “教堂-church” and “教宗-Pope”. Similarly, homophonic morphemes are common in the auditory modality. For example, the syllable/xin1/is the “heart” morpheme in/xin1tong4/(“心痛-heartache”) but the “bitter” morpheme in/xin1ku3/(“辛苦-hard work”). Although a more complex lexical tone system should, in principle, reduce the need to represent different meanings with the same sound, the problem does not completely disappear even in Chinese dialects with such systems. For example, Cantonese has six tones, but there are still many homophonic morphemes (such as/fu1/and/si1/, which are mapped to 58 and 54 characters, respectively). This one-to-many mapping is referred to as morphemic ambiguity [8, 9] and is analogous to “stick” in “sticky vs. chopstick” or “-er” in “teacher vs. taller”. Although morphemic ambiguity is a universal phenomenon that can be found in languages with varying degrees of morphological richness (e.g., English, Finnish, and Spanish), morphemic ambiguity is much more prevalent in Chinese. Such ambiguity reduces morphological decomposition efficiency because resource-demanding disambiguation is needed to discover the correct morphemic meanings. Given the high speed of language comprehension, Packard [6] suggested that Chinese words should instead be recognized holistically.

Whether Chinese words are processed holistically or through morphemes has been empirically tested in previous studies. In a series of experiments that examined word spacing in Chinese, Hsu and Huang [10, 11] revealed several pieces of evidence for the importance of words in Chinese language processing. First, in a read-aloud task, Chinese speakers paused longer between words than between characters/morphemes. Second, inserting spaces between characters, thereby disrupting the “wholeness” of words, led to longer reading times. Third, inserting spaces between words, which (in Chinese text) are conventionally unspaced, improved reading speed. In another study, Perea and Wang [12] examined the issue by comparing the performance in reading mono-colored text and multi-colored text that displayed words in alternating colors. The authors showed that the multi-colored text improved the read-aloud speed of grade-two children and of adult readers who were asked to read a difficult text, although no improvement was evident when adults read a simple text. The results suggest that providing word segmentation cues may facilitate performance in Chinese reading.

The decomposition vs. holistic views of Chinese word recognition have also been investigated using the eye-tracking technique (see Tsai & McConkie [13] and Tsang & Chen [14] for discussion). Unlike the results of studies of morphologically rich languages (e.g., Finnish and Uighur), where there is good evidence that morphemes are important in determining eye fixation locations [15, 16], the results of studies of Chinese have been equivocal. For example, following Hsu and Huang [10, 11], Bai, Yan, Liversedge, Zang, and Rayner [17] examined how word spacing affected the reading of Chinese text. The authors compared the effects of inserting extra physical spaces between words and between characters on eye movement. The results showed that inserting extra spaces between words did not affect fixation times but inserting extra spaces between characters produced interference. The authors attributed this performance decline to a disruption of word perception when extra spaces are inserted between characters. This disruption was taken as indirect evidence that despite the salience of characters, words remain the basic unit in Chinese reading.

Utilizing the eye tracking technology, Zhou, Wang, Shu, Kliegl and Yan [18] examined how providing color cues for word segmentation influenced eye movement when reading Chinese. In addition to the multi-colored word conditions, the authors introduced multi-colored nonword conditions, in which the colors alternated at positions not indicative of word boundary. Consistent with the results obtained by Perea and Wang [12], the results highlighted the importance of words. In particular, Zhou et al. [18] showed that the overall reading speed decreased in the nonword-marked condition. Moreover, when words were marked, readers tended to fixate on the center of words, which was in principle more informative. A recent eye tracking study found more robust benefits among Chinese children when explicit word boundary information afforded by color cues [19]. In another eye-tracking study, Yan, Tian, Bai, and Rayner [20] showed that although word frequency robustly affected fixation times when reading Chinese, character frequency mattered only when reading low frequency words. This discovery indicates that readers routinely processed Chinese words holistically. Comprehension through morphological decomposition was only a spare mechanism used when reading unfamiliar words.

While the aforementioned studies supported words as the major unit of processing in reading Chinese, Chen et al. [4] reached a different conclusion using the same eye-tracking technique. The authors adopted a regression approach in analyzing fixation times when reading Chinese. The results show that although word-level variables could account for more variances in reading times than morpheme-level variables among second graders, the reverse was true for sixth graders and undergraduate students. Based on these findings, the authors suggested that morphemes play a stronger role in Chinese word recognition for more proficient readers. In another eye-tracking study, Yen, Tsai, Tzeng, and Hung [21] showed that Chinese readers might be able to extract morphological information from a parafoveal preview. Specifically, fixation times on target words (e.g., “戒菸-to quit smoking”) were numerically shorter when readers received the same morpheme preview (e.g., “戒除-to quit/give up a habit”) compared to a different morpheme previews (e.g., “戒備-to guard against”).

Evidence for morphological decomposition during Chinese word processing can also be found in priming experiments. For example, Zhou et al. [5] manipulated the prime-target relationship (morphology, orthography, phonology, or semantics). The authors showed that morpheme-sharing primes (e.g., “华丽-magnificent”/hua2li4/) could facilitate target word recognition (e.g., “华贵-luxurious”/hua2gui4/). Moreover, such facilitation could not be explained by sharing lexical-level orthography, phonology, or semantics because these conditions produced effects that differed from the morphological condition in terms of either strength or time course. Tsang and Chen [9, 22] observed similar morphological priming effects while systematically examining the influences of morphemic ambiguity on morphological processing. The authors showed that morphological priming could be found regardless of the correct morphemic meanings of homographic morphemes, but stronger effects were observed for the more frequently used meaning. For example, “教” produced more robust priming when it was used as the more frequent “education” morpheme than when it was used as the less frequent “religion” morpheme. Therefore, contrary to what Packard [6] proposed, morphemic ambiguity did not always prevent morphological decomposition in Chinese word recognition.

In another masked priming experiment [23], participants performed lexical decisions on Chinese transparent bimorphemic target words (e.g., “闪光-flash”, which literally means “to flash-light”) that were preceded by opaque primes (e.g., “雷达-radar”, which literally means “thunder-reach”) or unrelated primes (e.g., “饭盒-lunchbox”, which literally means “rice-box”). The results show that, compared to unrelated conditions, responses were faster in opaque conditions. Because the opaque primes were related to the targets only through the first constituent morpheme meanings (primes and targets were unrelated on all dimensions at the lexical level), facilitative priming can be attributed only to meaning activation in opaque word constituents. Similarly, in English, effects cannot be attributed to orthographic sharing [24]. Morphological activation in opaque words provide strong evidence for obligatory morphological decomposition [25] because, in principle, opaque words can be understood only through holistic processing, as their constituents do not contribute to word meanings.

While accumulated evidence tends to support morphological decomposition during Chinese visual word recognition, at least for proficient language users [4], there are reasons to expect more holistic processing in spoken word recognition. First, as aforementioned, homophonic morphemes are common in Chinese. There are approximately 1200 and 1800 possible syllable-tone combinations in Mandarin and Cantonese, respectively; these combinations are used to represent over 6500 commonly used Chinese characters. These syllable-tone combinations increase the degree of morphemic ambiguity in Chinese speech, thereby possibly further reducing the efficiency of morphological processing [6]. Second, priming experiments in English suggest that morphological priming may be less robust in the auditory modality. Specifically, although opaque words were decomposed to produce facilitative priming in visual word recognition [25], similar facilitation was not found in spoken word recognition; the lack of facilitative priming indicates holistic processing of spoken opaque words [26].

Indeed, an early study showed that morpheme frequency did not affect Chinese spoken word recognition, but the effect of word frequency remained robust (Experiment 1, Zhou & Marslen-Wilson [27]). This finding suggests that morphological decomposition did not occur in the auditory modality. However, subsequent experiments that manipulated syllable frequency showed an unexpected inhibitory effect (i.e., higher syllable frequency led to slower word recognition). Accordingly, the authors speculated that morphemes were activated during Chinese spoken word recognition, but the facilitative effect of high morpheme frequency was offset by competition between homophonic morphemes (this competition led to a high syllable frequency).

Evidence for such competition was observed directly in a visual-world experiment by Tsang and Chen [8]. In a typical visual-world experiment, participants are given a spoken stimulus (e.g., a word or sentence) and a visual display that contains several objects in each trial. The eye movements of the participants on different objects of the visual display are monitored as the participants hear the spoken stimuli. By a simple linking hypothesis, the proportions of fixations on different objects reflect the activation levels of lexical representations underlying these objects (i.e., more fixations mean stronger activation). For example, Allopenna, Magnuson and Tanenhaus [28] showed that when hearing the word “beaker”, participants initially fixated more on “beaker” and “beetle” in the visual display; this fixation was taken as evidence of the coactivation of word candidates that matched with the input acoustic signals.

In Tsang and Chen [8], participants were given disyllabic Chinese spoken words. Each visual display contained three objects, two of which shared homophonic morphemes (e.g., “蜂蜜-honey”/**feng1**mi4/and “风筝-kite”/**feng1**zheng1/). Both objects were fixated on more often than the third, unrelated objects. Moreover, the more frequently used morphemes were fixated on more often than the less frequent morphemes before disambiguation. In second syllables, the proportions of fixations on incorrect objects decreased after disambiguation. However, move-in fixations were slower for more frequently used morphemes. These findings align with the proposal of competition among homophonic morphemes during Chinese spoken word recognition [27], thereby suggesting that more frequent morphemes in the homophonic set are more strongly activated and exert stronger competition than other less frequent morphemes. Overall, these preliminary results appear to support morphological decomposition in Chinese spoken word recognition. However, not as much empirical research has been conducted on auditory modality as on visual modality. It is important to obtain converging evidence before conclusively developing well-informed Chinese morphological processing theories.

This study’s goal is to further investigate morphological processing in Chinese spoken word recognition. As a morphologically impoverished language, the results of studies of Chinese provide an excellent contrast to the results of studies of morphologically rich languages, such as Finnish and Uighur [15, 16], where morpheme effects are strong. This comparison is important in developing more universal morphological processing theories. Specifically, two cross-modal priming experiments were conducted. The priming paradigm has been useful in examining morphological processing. This paradigm can provide converging evidence of the manipulation of morpheme frequency [27] and the visual-world paradigm [8]. Moreover, this study extended previous work in three directions. First, following Tsang and Chen [23], opaque words were used to provide a more rigorous test of morphological activation. Specifically, disyllabic opaque words served as auditory primes. Visual target words were semantically related to second morphemes in opaque or whole opaque words. The word-related targets allowed us to compare morphological and lexical priming effects. In previous visual experiments, first morphemes were tested instead of second morphemes. However, given that speech signals unfold over time, disyllabic word recognition is possible only when information about second syllables/morphemes become available. Therefore, the present auditory experiments focus on second morphemes to allow a fair comparison between morphological and lexical effects. Each related condition had a corresponding unrelated baseline. If Chinese spoken words are recognized through morphological decomposition, facilitative priming should be observed when prime-target relatedness is established through second constituent morpheme meanings.

Second, we tested whether morphological activation could be eliminated by prior context. Spoken opaque primes were preceded by sentence contexts that were either neutral or biased toward whole opaque word meanings. Previous studies of spoken word processing tested the processing of isolated words [8, 27]. However, in natural language use, word recognition is never an isolated event but occurs under the influence of various contextual factors, such as semantic constraints imposed by the text, the reader’s prior knowledge, and pragmatic considerations. It is well-established that sentence contexts can modulate lexical and sublexical processing in both the visual and auditory modalities [29, 30, 31]. If contextual constraints also modulate morphological processing, morphological priming might be observed only in the neutral context but not when the sentence is biased toward whole opaque word meanings. Such contextual modulation might also explain why comprehension could remain highly efficient despite the evidence of morphological decomposition in isolated word recognition and the prevalence of morphemic ambiguity in Chinese [6].

Third, visual targets were presented either at the onset of second syllables in opaque prime words or at the onset of subsequent syllables in carrier sentences. This pattern allowed us to examine the time course of morphological and lexical activation. Visual instead of auditory targets were used because the opaque words were embedded in the middle of the spoken sentences (to prevent the influences of sentence wrap-up effects when the words were embedded at sentence end). Therefore, it would be impossible to present auditory targets clearly to the participants. If Chinese spoken words are recognized through morphological decomposition, morpheme meanings should be activated before word meanings. Accordingly, morphological and lexical priming should be significant at the onset of second and subsequent syllables, respectively. Moreover, if the biasing context can enhance whole-word meaning availability, lexical priming should be observed earlier in the biasing context than in the neutral context condition, as indicated by significant lexical effects at the onset of second syllables. This study’s design and sample materials are shown in Table 1.

**Table 1:** Example materials in Experiment 1 (neutral context) and Experiment 2 (biasing context).

Sentence	Target condition	Target word	Word frequency in millions	Number of strokes
她终于感觉我的**刘****海**自然多了。 She finally feels that my **fringe of hair across the forehead** looks more natural.	Morpheme-related	湖泊 (lake)	70.85 (35.76)	16.42 (0.83)
	Morpheme-unrelated	筋骨 (bones)	67.88 (33.07)	16.77 (0.69)
理发师把垂在我额头上的**刘****海**修剪得很漂亮。 The barber helps me cut my **fringe of hair across the forehead** beautifully.	Word-related	发型 (hairstyle)	36.32 (11.8)	15.04 (0.79)
	Word-unrelated	耐心 (patience)	39.67 (11.8)	15.85 (0.79)

The critical opaque words (**刘海**) mean “fringe of hair across the forehead,” but the literal meaning is “Liu (a surname)-sea”. The opaque prime words are bolded in the sentences. The target words appeared either at the onset of the second syllable of opaque words (e.g., “海”) or at the onset of the subsequent syllable in the sentences (e.g., “自” or “修”; underlined in the examples). For word frequency and number of strokes, means and standard errors (in brackets) are provided.

## Experiment 1: Neutral context

In Experiment 1, auditory opaque prime words were embedded in neutral sentences. Visual targets were semantically related to either second constituent morphemes in opaque or whole opaque words to reveal morphological and lexical priming, respectively. In addition, targets were presented either at the onset of second syllables in opaque primes or at the onset of subsequent syllables in sentences to examine the time course of morphological and lexical activation. It was predicted that morphological priming would be observed when targets were presented at the onset of second syllables, while lexical priming would be observed when targets were presented at the onset of subsequent syllables after prime words.

### Materials and design

Twenty-six Chinese bimorphemic opaque words were chosen from a pool of 160 words after a series of pilot tests. First, 20 undergraduates rated the semantic relatedness of second morphemes and whole opaque words on a 5-point Likert scale (a higher score = stronger relatedness). The average relatedness for the chosen materials was low (mean = 1.93, standard deviation (S.D.) = .38), thus confirming their semantic opacity. Next, the second morphemes of nine opaque words had homophones in Mandarin. However, we used the most frequent meanings, as shown in a pilot test that asked 20 undergraduates to write down the first character they thought of after hearing homophonic morphemes presented in isolation (the mean report rate of the morphemes chosen was 89%). Third, to ensure that the participants could not predict the whole opaque words by just hearing the first morphemes, 20 undergraduates were asked to write down the first two-character word that they thought of after hearing the first morphemes presented in isolation. The mean report rate of the opaque words chosen was 3%.

Sentence contexts were then constructed for the opaque words. The sentences contained from 13 to 23 syllables (mean = 17.27, S.D. = 2.50). The opaque words appeared roughly in the middle of the sentences. The sentences were neutral to the opaque words, as shown in a cloze probability test that asked 32 undergraduates to write down the first word they thought of after reading the sentences preceding the opaque primes. The opaque words chosen were never reported (i.e., cloze probability = 0). The materials were then recorded by a female native Mandarin speaker. The mean durations of the entire sentences and the critical second morphemes were 9571.20 ms (S.D. = 1077.60) and 556.77 ms (S.D. = 99.43), respectively.

Four bimorphemic targets were prepared for each opaque prime. The first target was related to the second morpheme’s meaning in the opaque word, and the second target was the corresponding unrelated control. The third target was related to the entire opaque whole-word meaning, and the fourth was the word-unrelated control. The frequency and number of strokes between the related and unrelated targets (morpheme-related vs. morpheme-unrelated targets, and word-related vs. word-unrelated targets) were matched (*p*s > .1). The semantic relatedness between second morphemes in opaque primes and four targets, as rated by 57 participants on a 5-point Likert scale (higher score = stronger relatedness), was higher in the morpheme-related condition than in the morpheme-unrelated, word-related and word-unrelated condition *(ps* < .001). Similarly, the semantic relatedness between whole opaque prime words and their targets was higher in the word-related condition than in the word-unrelated, morpheme-related and morpheme-unrelated condition (*ps* < .001). Finally, the opaque words and the targets were unrelated in visual and auditory forms to prevent orthographic and phonological priming.

Because the target type was a between-item manipulation, a pilot experiment was conducted to ensure that different targets led to comparable lexical decision performance when presented in isolation. For morpheme-related and morpheme-unrelated conditions, the reaction times (598 ms vs. 601 ms, respectively) and accuracies (97% vs. 97%, respectively) were closely matched. Similarly, the reaction times (623 ms vs. 620 ms) and accuracies (98% vs. 96%) of word-related and word-unrelated conditions, respectively, were also matched. The difference in reaction times between morphemes and word conditions was also nonsignificant (*p* = .17). This pilot experiment helped ensure that any effects obtained in the primary experiment could not be attributed to differences in targets across conditions.

In addition to target type and relatedness, the experiment also included target onset (second syllable vs. subsequent syllable) as a factor. Because only 26 opaque words were obtained during pilot testing, manipulating and counterbalancing all factors within-subject would leave only a few items per condition. To solve this problem, target type and target onset were manipulated between-subject, while relatedness was manipulated within-subject. Although a complete within-subject design is preferred in psycholinguistic experiments, random participant assignment to experimental conditions may help eliminate participant biases in between-subject design. Four experimental lists were constructed. The first and second lists contained morpheme-related and morpheme-unrelated targets presented at second syllable and subsequent syllable onset, respectively. Similarly, the third and fourth lists contained word-related and word-unrelated targets presented at second syllable onset and subsequent syllable onset, respectively. Furthermore, two versions were created for each list to counterbalance related and unrelated prime-target pairing, thereby resulting in eight lists in total. The same set of 50 filler sentences was added to each list. Twelve filler sentences were paired up with unrelated word targets presented visually at different points in the sentences, thereby reducing participants’ awareness of manipulation (only 34% of the items had related prime-target pairing). Thirty-eight fillers were paired up with nonword targets created by noninterpretably combining two real Chinese morphemes for the lexical decision task. To summarize, each list contained 76 items, including 13 related and 13 unrelated experimental targets, 12 filler words, and 38 nonword targets. These items were presented for each participant at random.

### Procedure

Each trial started with a fixation cross presented for 1000 ms in the center of the computer screen. The auditory sentence was then played via headphones at a comfortable volume level adjusted for each participant. The visual target appeared for 500 ms either at the onset of the second morpheme in the opaque word or at the onset of subsequent syllables. Participants were instructed to decide as quickly and as accurately as possible whether the target was a real Chinese word or not. In 20 filler sentences, a multiple-choice comprehension test was provided at the end of the trial to encourage participants to pay attention to sentence comprehension. The entire experiment lasted for 30 minutes.

### Participants

We recruited 164 undergraduate students from South China Normal University. Although the students spoke different dialects, the students had all received formal education in Mandarin Chinese since primary school. Mandarin Chinese was also the primary language they used in daily life and in school. Therefore, the students were considered proficient Mandarin Chinese speakers. The students had normal hearing and normal or corrected vision. Participants were randomly assigned to the eight experimental lists. Data from 4 participants were discarded for excessive errors in the lexical decision task; consequently, each of the eight experimental lists contained 20 participants. This study was approved by the ethics committee of the School of Psychology, South China Normal University. Participants were required to read and sign the consent form before the experiment and were paid 25 RMB after the experiment.

### Results and discussion

Errors and trials with reaction times larger than 3 S.D.s from individual means (total 4.71%) were discarded from further analyses. Table 2 displays the mean reaction times and accuracy of the remaining data in different conditions. As shown in the table, the priming effect was quite clear between morpheme-related and morpheme-unrelated conditions, while the effect was much weaker in word-related versus word-unrelated conditions. To statistically evaluate the observed patterns, we conducted a linear mixed model (LMM) for reaction times and a generalized linear mixed model (GLMM) for accuracy. Since word frequency was not matched between morpheme and word conditions, we conducted 2 * 2 analysis for the morpheme and word condition with relatedness (related vs. unrelated), target onset (second syllable vs. subsequent syllable) and their interaction as fixed factors. All fixed effects were effect coded, with the first listed level of each variable coded as 0.5 and the second as -0.5. We followed Barr, Levy, Scheepers and Tily [32] in using the maximal random effect model. Additionally, because we used a between-subject design in the present study, the random slope on participants could not be estimated [32]. Thus, the maximal random effect model in the following analysis included the random intercept on participants and items, the random slope for the relatedness, the target onset and their interaction on items. Furthermore, since this study focuses mainly on the priming effect (relatedness effect), we conducted pairwise comparisons on different target types at different target onsets until we found a significant main effect or interaction.

**Table 2:** Mean reaction times (ms) and accuracies on different conditions in Experiment 1 (N = 160; S.D. in brackets).

		Morpheme-related	Morpheme-unrelated	Priming effect	Word-related	Word-unrelated	Priming effect
At second morpheme onset	Reaction times	769.81 (222.96)	808.62 (237.16)	38.81**	766.48 (188.00)	763.90 (182.21)	-2.58
	Accuracies	.97 (.05)	.97 (.06)	.00	.97 (.05)	.97 (.05)	.00
At subsequent syllable onset	Reaction times	747.71 (183.19)	789.83 (203.46)	42.12*	764.02 (205.58)	766.05 (184.51)	2.03
	Accuracies	.96 (.06)	.96 (.05)	.00	.99 (.03)	.95 (.06)	-.04**

*: *p* < .05;

**: *p* < .001.

When we analyzed accuracies on the morpheme condition, all effects were nonsignificant. However, in analyzing the word condition, the effects of relatedness and relatedness by target onset interaction were significant (see Table 3). Further planned comparisons indicated that responses were more accurate in the word-related rather than the word-unrelated conditions when the targets were presented at subsequent syllable onset (*Estimate* = 1.60, *SE* = .46, *Z* = 3.52, *p* < .001).

**Table 3:** The main and interaction effect in Experiment 1.

		Effect	Estimated	*SE*	*t*	*p*
Morpheme condition	Accuracies	Relatedness	0.03	0.42	0.08	0.93
		Target onset	0.61	0.48	1.27	0.21
		Relatedness*Target onset	-0.27	0.86	-0.31	0.75
	Reaction times	Relatedness	-42.44	13.58	-3.13	0.005
		Target onset	21.89	29.33	0.75	0.46
		Relatedness*Target onset	3.41	14.89	0.23	0.82
Word condition	Accuracies	Relatedness	0.78	0.34	2.28	0.02
		Target onset	-0.38	0.38	-1.01	0.31
		Relatedness*Target onset	-1.72	0.68	-2.52	0.01
	Reaction times	Relatedness	-3.22	13.20	-0.24	0.81
		Target onset	0.17	24.14	0.01	0.99
		Relatedness*Target onset	-2.63	16.76	-0.16	0.88

Analyzing reaction times in the morpheme condition showed that only the effect of relatedness was significant. In the word condition, all the effects were nonsignificant (see Table 3). Further pairwise comparisons indicated that responses were faster in the morpheme-related than in the morpheme-unrelated condition when the targets were presented both at second syllable onset (*Estimate* = -40.15, *SE* = 11.13, *t* = -3.60, *p* < .001) and at subsequent syllable onset (*Estimate* = -43.54, *SE* = 16.82, *t* = -2.59, *p* = .02).

The results of Experiment 1 showed that when the prior context did not constrain opaque prime words, facilitative priming was observed only when the targets were related to second morpheme meanings in opaque prime words. This effect appeared relatively early at the second syllable onset and persisted to the subsequent syllable onset. In contrast, whole opaque word meanings were activated relatively late, thus producing significant facilitation only at subsequent syllable onset. Although it was unclear why the morphological effect emerged in reaction times while the lexical effect was found only in accuracies, the morpheme-then-word sequence of priming effects showed that morphological activation preceded lexical activation, thus being consistent with the morphological decomposition hypothesis during Chinese spoken word recognition.

## Experiment 2

Experiment 2’s design was identical to Experiment 1’s, except that the opaque prime words were embedded in sentences that were biased toward whole opaque word meanings. Comparing these two experiments’ results made it possible to investigate whether sentential supports would reduce or eliminate morphological activation and lead to holistic opaque word processing. It was predicted that when prior context was available to support whole opaque words, lexical priming would emerge earlier at the onset of the second syllable.

### Materials, design and procedure

The methodological details were identical to those in Experiment 1, except that two opaque words were removed because we could not construct appropriate biasing in sentence contexts that fulfilled the pilot test requirements (two nonword items were also removed to balance the number of yes-no responses). The remaining items’ cloze probability was high (mean = 80%, S.D. = 17%), thus indicating strong sentential biases. The sentences contained 17 to 25 syllables (mean = 19.58, S.D. = 2.0), and opaque words appeared roughly midsentence. The mean durations of entire sentences and critical second morphemes were 9111.88 ms (S.D. = 922.33) and 552.21 ms (S.D. = 68.83), respectively.

### Participants

We recruited 167 undergraduate native Mandarin Chinese speakers from South China Normal University. The participants had characteristics similar to those of the participants in Experiment 1 but had not participated in other pilot tests or Experiment 1. The participants were randomly assigned to the eight lists. Data from 7 participants were discarded for excessive errors in the lexical decision task, thus leaving 20 participants in each of the eight experimental lists.

### Results and discussion

Errors and trials with reaction times greater than 3 S.D.s of individual means (total 4.9%) were discarded from further analyses. Table 4 displays the mean reaction times and accuracies of the remaining data in various conditions. Analyses were conducted as in Experiment 1.

**Table 4:** Mean reaction times (ms) and accuracies (%) on different conditions in Experiment 2 (N = 160; S.D. in brackets).

		Morpheme-related	Morpheme-unrelated	Priming effect	Word-related	Word-unrelated	Priming effect
At second morpheme onset	Reaction times	750.13 (225.36)	755.81 (202.29)	5.69	679.30 (176.12)	714.24 (169.18)	34.94*
	Accuracies	.98 (.04)	.97 (.05)	-.01	.96 (.05)	.94 (.08)	-.02
At subsequent syllable onset	Reaction times	712.21 (162.54)	713.28 (169.50)	1.07	725.64 (181.68)	728.70 (183.09)	3.06
	Accuracies	.97 (.06)	.97 (.05)	.00	.96 (.08)	.95 (.08)	-.01

*: *p* < .05.

All effects on the accuracies on the morpheme and word condition were nonsignificant (see Table 5).

All effects on the reaction times on the morpheme condition were nonsignificant. However, regarding the word condition, the effect of relatedness and relatedness by target onset interaction were significant (see Table 5). Further planned comparisons indicated that responses were faster in the word-related than in the word-unrelated condition when the targets were presented at second syllable onset (*Estimate* = -34.56, *SE* = 10.34, *t* = -3.34, *p* = .002), although the effect disappeared quickly at subsequent syllable onset (*p* >.1).

**Table 5:** The main and interaction effect in Experiment 2.

		Effect	Estimated	*SE*	*t*	*p*
Morpheme condition	Accuracies	Relatedness	-0.16	0.48	-0.34	0.74
		Target onset	0.39	0.48	0.82	0.41
		Relatedness*Target onset	-0.06	0.94	-0.06	0.95
	Reaction times	Relatedness	-1.42	10.11	-0.14	0.89
		Target onset	37.56	29.93	1.26	0.21
		Relatedness*Target onset	-7.03	13.29	-0.53	0.60
Word condition	Accuracies	Relatedness	0.32	0.34	0.96	0.34
		Target onset	-0.20	0.40	-0.51	0.61
		Relatedness*Target onset	0.66	0.68	0.96	0.34
	Reaction times	Relatedness	-20.60	8.61	-2.39	0.03
		Target onset	-33.90	25.12	-1.35	0.18
		Relatedness*Target onset	-28.62	14.41	-1.99	0.05

When the prior context supported whole opaque words, their second morpheme meanings no longer facilitated target recognition, as in the neutral context. In contrast, whole opaque word meanings activated more rapidly, thereby producing facilitation at the onset of the second syllable in opaque primes (as opposed to subsequent syllable onset in Experiment 1). Moreover, the effect was significant in the reaction time analysis (as opposed to the accuracy analysis in Experiment 1). This finding supports that context can modulate morphological processing during Chinese spoken word recognition.

## Discussion

This study adopted the cross-modal priming paradigm to investigate morphological processing in Chinese spoken word recognition. Disyllabic (bimorphemic) opaque words served as auditory primes. These opaque primes were embedded in sentences that were either neutral (Experiment 1) or biased toward whole opaque word meanings (Experiment 2). Visually presented target words were semantically related to either whole opaque primes or second constituent morphemes in opaque primes. Each related target had its corresponding unrelated control target to evaluate facilitative priming strength. The target words were presented either at the onset of the second syllables in opaque primes or at the onset of subsequent syllables in sentences to examine the relative timing of morpheme and whole-word activation.

The results showed that, in a neutral context, facilitative priming was found in both the morpheme-related and word-related conditions. However, the morpheme effect emerged earlier than the word effect, thereby supporting the morphological decomposition hypothesis. In contrast, when prior sentence contexts that were biased toward whole opaque word meanings were available, morphological priming disappeared, while lexical priming remained significant. Moreover, compared to a neutral context, the whole-word effect emerged earlier in the biased context condition. Specifically, the effect was found at the onset of second syllables in opaque primes (i.e., before all perceptual features of the opaque words were available). These findings suggested that context can modulate morphological processing.

### Morphological decomposition in spoken word recognition

This study contributes extensively to understanding morphological processing in Chinese spoken word recognition. First, Experiment 1’s results (neutral context) provide converging evidence of morphological decomposition, which was observed primarily in visual word recognition [5, 9, 22]. Extending morphological processing to spoken Chinese is particularly interesting because, in principle, the prevalence of morphemic ambiguity should have made decomposition highly inefficient [6]. That morphemes are still activated indicates that multiple factors drive morphological processing. For example, influences on morphemic ambiguity in Chinese might have been counteracted by effortlessly detecting morpheme boundaries (i.e., detecting spaces and syllable onset in the visual and auditory modalities, respectively). Similarly, in morphology-rich languages, morphological processing may be triggered by detecting frequently occurring affixes, thereby allowing effortless affix stripping [33].

Second, Experiment 1 also showed that morphological priming emerged earlier than lexical priming. This sequence provides strong evidence that morphological activation is a prelexical event—activated morphemes serve as access codes to lexical representations [25, 34]. In contrast, the morpheme-then-word sequence is inconsistent with models that assume decomposition after lexical access [35]. Moreover, supralexical models typically assume that morphemes are used to retrieve word meanings. Therefore, decomposition is expected only for transparent words with meanings that can be predicted from constituent morphemes. In other words, supralexical models also fail to explain morphological priming observed in opaque Chinese words.

Third, this study also has greater implications in understanding the roles of morphemic form and morphemic meaning in word recognition. Based on previous masked priming experiments [25, 36], it is now widely accepted that morphological decomposition depends only on surface form; therefore, all words that appear morphologically complex will be decomposed into constituent morphemes for recognition regardless of whether the morpheme and word meaning match (see Feldman, O’Connor, & Moscoso del Prado Martín [37] for an alternative view). On the other hand, it is less clear whether decomposed morpheme meanings are simultaneously activated upon decomposition or whether the decomposed morphemes serve only to access lexical representations, which in turn, retrieve meanings holistically. In this study, prime-target pairs of the morphological condition were related only through second constituent morpheme meanings in opaque words (see Table 1). Nevertheless, morphological priming was observed in a neutral context. Combined with similar findings in visual word recognition [23], the results suggest that morphemic meanings are indeed activated in opaque word recognition, even when these meanings are inconsistent with whole-word meanings.

Why are morpheme meanings activated in opaque word recognition even when they do not contribute to word meanings? The answer may be that morphological decomposition helps word recognition most of the time. Most words are transparent, while opaque or pseudocomplex words are exceptions. In other words, morpheme meanings usually contribute to word meanings. Therefore, it is conceivable that the language system would generalize a mechanism that is usually effective and, in rare cases, generates errors. This idea has received support from a recent computer simulation [38] that showed a model that assumes both decomposition based on morphemic form and immediate morphosemantic activation of decomposed morphemes; this model can most accurately explain various empirical findings. The model also indicates that some opaque words would be read transparently (e.g., “corner” as “corn grower”), at least temporarily. Such errors occurred precisely because morphosemantic activation helps retrieve lexical meanings most of the time, thereby making “transparent reading” the default mechanism of word recognition even when, in some rare cases, the reading turns out to be an inaccurate.

On the other hand, some studies also fail to observe morphosemantic activation for opaque words. For example, Zwitserlood [39] compared the priming effects produced by transparent and opaque words in two priming experiments. In Experiment 1, targets were the constituent morphemes of primes (i.e., partial repetition priming, e.g., “teaspoon-TEA” and “buttercup-BUTTER”). In Experiment 2, primes and targets were related through semantic association of the constituent morphemes (i.e., semantic associate priming, e.g., teaspoon-COFFEE” and “buttercup-BREAD”). Prime words were presented for 200 ms, followed by a blank screen of 100 ms and the targets for 400 ms. The results show that both the transparent and opaque primes produced significant partial repetition priming, but only the transparent primes produced semantic priming. Similar results were obtained in Sandra [40] with a modified priming paradigm. In contrast to the typical priming procedure, in which participants respond only to target words, the modified procedure required responses to both primes and targets. Each word was presented 240 ms after a response to the previous word had been made. Again, facilitative priming was found only in the transparent condition and not in the opaque condition. These findings indicate that constituent morpheme meanings were not activated for opaque words and were contrary to the results of this study, Marelli and Baroni [38], and Tsang and Chen [23].

The discrepancy among studies might be related to language differences. The materials in both Sandra [40] and Zwitserlood [39] were Dutch, which has a more complicated inflectional system than Chinese (this study; Tsang & Chen [23]) and English [38]. Because inflection typically does not change stems’ core meanings, Dutch users might focus more on the morphemic form/structure and devote fewer resources to retrieving morpheme meanings. Additionally, differences in experimental procedures might also have contributed. In Sandra [40] and Zwitserlood [39], visually presented prime word durations were much longer than 40 ms, as in Tsang and Chen [23]. Therefore, the morphosemantic effects observed in Tsang and Chen [23] might reflect an early processing stage, while the results of Sandra [40] and Zwitserlood [39] might reflect a later stage. In this study, primes were presented auditorily; an auditory presentation cannot be directly compared with a visual presentation. However, considering that the targets were presented either at the onset of the second syllables in the opaque primes or at the onset of the subsequent syllables after the opaque primes, it was likely that the results also reflect a relatively early stage of spoken word recognition. Moreover, computer simulations by Marelli and Baroni [38] also indicate that constituent morpheme meanings in opaque words are activated only in the early stage. Together, these results indicate that more research should examine the validity of morphosemantic activation across different languages and the time course of such activation. In particular, ERP recording would be the ideal tool for examining the time course of morphological processing because of the superior temporal resolution of ERP recording over behavioral measures.

### Contextual modulation on morphological processing

Another major contribution of this study is that it provides empirical evidence showing that context can modulate morphological processing. Many previous morphological processing studies were conducted with isolated words. Only a few have addressed the issue in a sentence’s context. However, context is important not only because it is an integral part of natural language use but also because psycholinguistic research in other areas has shown the robust influence of context on lexical and sublexical processing. In this study, the context effect revealed as the morphosemantic priming observed in a neutral context (Experiment 1) disappeared when prior sentence contexts were strongly biased toward whole-word meanings (Experiment 2). Such context effects may explain why typical language users are unaware of comprehension challenges when morphemic meanings are inconsistent with whole-word meanings (as in the cases of homographic/homophonic morphemes and opaque words). When there are sufficient contextual supports, whole-word meanings can easily be activated, thereby counteracting (incorrect) morphemic meanings.

The sentence context might modulate morphological processing in several ways. First, participants may predict critical opaque words before they are encountered, thereby completely bypassing bottom-up morphemic inputs. This occurrence is especially probable in this study, given the strongly biasing context used. Second, morpheme meanings and word meanings might be in parallel but independent races, as assumed in dual-route models [41]. Holistic word recognition may be completed rapidly with the help of biasing context before morphemes are activated strongly enough to influence processing. Third, the morpheme-route and holistic-route might interact such that context may strongly activate word meanings; this activation, in turn, may suppress morpheme activation. Future research is needed to verify these possible mechanisms.

Compared with other studies that investigated morphological processing in a sentence context, the context effect in this study appears to play a more determining role. For example, employing the eye-tracking technique, Juhasz [42] examined the morpheme frequency effect of English bimorphemic compound words (e.g., “flashlight”) in neutral and biasing sentences. In a neutral context (e.g., “It is unfortunate that you broke the expensive **flashlight** that Jerry gave us”), it was shown that higher frequency morphemes led to faster reading times in both early and late eye movement measures. However, when context provided constraints toward the target words (e.g., “The power went out and I do not have batteries for my **flashlight** or my radio”), the effect of first morphemes disappeared in first fixation durations and single fixation durations, although the effect reemerged in gaze durations and total reading times. Simultaneously, the effect of second morphemes remained robust throughout all eye movement measures. Therefore, while the results in a neutral context were consistent across studies in supporting morphological processing, the contextual constraint in the biasing context condition was more restricted in Juhasz [42].

In another eye-tracking study, Amenta, Marelli, and Crepaldi [43] made use of an interesting property of some Italian-derived words, namely, that the same word form can simultaneously have both a transparent and an opaque reading. For example, “copertina” can be read transparently as “small blanket” or opaquely as “cover”, depending on context. Amenta et al. [43] showed that when context demands transparent reading, morpheme frequency produces the typical facilitative effect on first fixation durations (i.e., higher frequency morphemes lead to faster reading). However, when context demands opaque reading, the morpheme frequency effect becomes inhibitory. The authors interpreted these results as supportive evidence for morphosemantic activation. In the transparent condition, the activated morpheme meanings were consistent with whole-word meanings, and the effect was, thus, facilitative. In the opaque condition, the morphemic meanings interfered with whole-word meanings, thereby producing an inhibitory effect. On one hand, these findings are consistent with current results in suggesting an early morphosemantic activation even for opaque words. On the other hand, these findings show that a context biased toward whole opaque words cannot eliminate morphological processing, thus deviating from the strong contextual constraints observed in Experiment 2 of this study.

Again, various factors might be responsible for the discrepancy in the strength of context effects across studies. First, Chinese is considered a highly context-dependent language [44]. For example, as aforementioned, the correct meanings of homographic or homophonic morphemes are determined by context. Similarly, because Chinese verbs do not inflect, their aspectual characteristics are revealed either by aspect markers (i.e., extra words) or by sentence context. Therefore, Chinese readers might be more sensitive to contextual effects during sentence comprehension. Second, the contextual bias of sentences used in this study was stronger than in previous studies. Specifically, the cloze probability of opaque prime words, given the sentence contexts, was 80% in Experiment 2, while cloze probabilities were approximately 70% in Juhasz [42] and below 35% in Amenta et al. [43]. The more constraining sentence contexts in this study might lead to stronger effects in processing. In other words, contextual strength appears to be a critical factor. Future research could manipulate contextual strengths directly to investigate this possibility.

In summary, we obtained evidence that morphemes play a role in Chinese spoken word recognition, even when the prevalence of morphemic ambiguity led to the opposing proposal that Chinese words should be recognized holistically [6]. Simultaneously, morphological processing is not “obligatory” in the strict sense. Morpheme activation was sensitive to context such that morpheme activation disappeared when context was strongly biased toward the meanings of whole words. Daily language is usually supported by a rich context; this support may explain why language users are not particularly troubled by the (inaccurate) morphosemantic activation of opaque words.

### Limitation

Ideally, experimental manipulations in psychology should be made within-subject and within-item. However, a common problem for many psycholinguistic studies is the limited number of possible items (i.e., words) after controlling for several potential confounding variables. Similarly, one caveat in understanding the results of this study was the relatively few opaque word items remaining after controlling for various factors (26 were chosen from an original pool of 160). As a result, a between-subject design was adopted to ensure that the condition mean of each participant would be reasonably stable. To be specific, each participant was presented with 13 related and 13 unrelated targets in one of the four conditions crossed by target type (morpheme vs. word) and target onset (second syllable vs. subsequent syllable). A between-subject design always had the potential of confounding individual difference. In this study, individual difference was partially counterbalanced by recruiting undergraduate students with a similar language background from the same university as participants and focusing on the priming effects in analyses (the effect of individual differences was at least partially reduced when comparing the related and unrelated targets). Similarly, the paucity of matched items made it impossible to include word frequency manipulation in the design, although there was evidence that morphemes might play a stronger role in low frequency words [20]. To solve the problems of material matching, future studies can adopt a megastudy approach. In contrast to traditional experiments, which have few items due to strict experimental control, a megastudy unrestrictedly includes thousands of items. Then, the effect of the variable of interest can be evaluated after statistically controlling for the effects of other variables in regression-based analyses. This approach is indeed becoming increasingly popular in psycholinguistic research [2] to complement factorial experiments. We believed it would be equally helpful in testing the decomposition vs. holistic view of Chinese word recognition.

## Supporting information

S1 Data (XLS)

## References

[1] Cai Q, Brysbaert M. SUBTLEX-CH: Chinese Word and Character Frequencies Based on Film Subtitles. PLoS ONE. 2010; 5: e10729 10.1371/journal.pone.0010729 20532192 PMC2880003

[2] Tsang YK, Huang J, Lui M, Xue M, Chan YWF, Wang S, et al MELD-SCH: A megastudy of lexical decision in simplified Chinese. Behav Res Methods. 2018; 50: 1763–1777. 10.3758/s13428-017-0944-0 28779457

[3] Hoosain R. Psychological reality of the word in Chinese. Adv Psychol. 1992; 90: 111–130.

[4] Chen HC, Song H, Lau WY, Wong KFE, Tang SL. Developmental characteristics of eye movements in reading Chinese. In McBride-Chang C, Chen HC editors. Reading Development in Chinese Children. Westport: Praeger Publishers; 2003. pp. 157–169.

[5] Zhou X, Marslen-Wilson W, Taft M, Shu H. Morphology, orthography, and phonology in reading Chinese compound words. Lang Cognitive Proc. 1999; 14: 525–565.

[6] Packard JL. Lexical access in Chinese speech comprehension and production. Brain Lang. 1999; 68: 89–94. 10.1006/brln.1999.2102 10433744

[7] Liu Y, Shu H, Li P. Word naming and psycholinguistic norms: Chinese. Behav Res Methods. 2007; 39: 192–198. 10.3758/bf03193147 17695344

[8] Tsang YK, Chen HC. Morphemic ambiguity resolution in Chinese: Activation of the subordinate meaning with a prior dominant-biased context. Psychon Bull Rev. 2010; 17: 875–881. 10.3758/PBR.17.6.875 21169583

[9] Tsang YK, Chen HC. Early morphological processing is sensitive to morpheme meanings: Evidence from processing ambiguous morphemes. J Mem Lang. 2013a; 68: 223–239.

[10] Hsu SH, Huang KC. Effects of word spacing on reading Chinese text from a video display terminal. Percept Mot Skills. 2000a; 90: 81–92. 10769885 10.2466/pms.2000.90.1.81

[11] Hsu SH, Huang KC. Interword spacing in Chinese text layout. Percept Mot Skills. 2000b; 91: 355–365. 11065294 10.2466/pms.2000.91.2.355

[12] Perea M, Wang X. Do alternating-color words facilitate reading aloud text in Chinese? Evidence with developing and adult readers. Mem Cognit. 2017; 45: 1160–1170. 10.3758/s13421-017-0717-0 28608193

[13] Tsai JL, McConkie GW. Where do Chinese readers send their eyes? In: Hyönä J, Radach R, Deubel H, editors. The Mind’s Eye: Cognitive and Applied Aspects of Eye movement Research. Amsterdam: Elsevier; 2003 pp. 159–176.

[14] Tsang YK, Chen HC. Eye movement control in reading: Logographic Chinese versus alphabetic scripts. PsyCh J. 2012; 1: 128–142. 10.1002/pchj.10 26272763

[15] Hyönä J, Yan M, Vainio S. Morphological structure influences the initial landing position in words during reading Finnish. Q J Exp Psychol. 2018; 71: 122–130. 10.1080/17470218.2016.1267233 27905866

[16] Yan M, Zhou W, Shu H, Yusupu R, Miao D, Krügel A, et al Eye movements guided by morphological structure: Evidence from the Uighur language. Cognition. 2014; 132: 181–215. 10.1016/j.cognition.2014.03.008 24813572

[17] Bai X, Yan G, Liversedge SP, Zang C, Rayner K. Reading spaced and unspaced Chinese text: evidence from eye movements. J Exp Psychol: Hum Percept Perform. 2008; 34: 1277–1287. 18823210 10.1037/0096-1523.34.5.1277 PMC2662925

[18] Zhou W, Wang A, Shu H, Kliegl R, Yan M. Word segmentation by alternating colors facilitates eye guidance in Chinese reading. Mem Cognit. 2018; 46: 729–740. 10.3758/s13421-018-0797-5 29435825

[19] Pan J, Liu M, Li H, Yan M. Chinese children benefit from alternating-color words in sentence reading. Reading and Writing. Forthcoming.

[20] Yan G, Tian H, Bai X, Rayner K. The effect of word and character frequency on the eye movements of Chinese readers. Br J Psychol. 2006; 97: 259–268. 10.1348/000712605X70066 16613652

[21] Yen MH, Tsai JL, Tzeng OJ, Hung DL. Eye movements and parafoveal word processing in reading Chinese. Mem Cognit. 2008; 36: 1033–1045. 10.3758/mc.36.5.1033 18630209

[22] Tsang YK, Chen HC. Morpho-semantic processing in word recognition: evidence from balanced and biased ambiguous morphemes. J Exp Psychol: Learn Mem Cogn. 2013b; 39: 1990–2001. 23834058 10.1037/a0033701

[23] Tsang YK, Chen HC. Activation of morpheme meanings in processing opaque words. Psychon Bull Rev. 2014; 21: 1281–1286. 10.3758/s13423-014-0589-2 24510473

[24] Fiorentino R, Fund-Reznicek E. Masked morphological priming of compound constituents. Ment Lex. 2009; 4: 159–193.

[25] Rastle K, Davis MH, New B. The broth in my brother’s brothel: Morpho-orthographic segmentation in visual word recognition. Psychon Bull Rev. 2004; 11: 1090–1098. 10.3758/bf03196742 15875981

[26] Marslen-Wilson W, Tyler LK, Waksler R, Older L. Morphology and meaning in the English mental lexicon. Psychol Rev. 1994; 101: 3–33.

[27] Zhou X, Marslen-Wilson W. Words, morphemes and syllables in the Chinese mental lexicon. Lang Cognitive Proc. 1994;.9: 393–422.

[28] Allopenna PD, Magnuson JS, Tanenhaus MK. Tracking the time course of spoken word recognition using eye movements: Evidence for continuous mapping models. J Mem Lang. 1998; 38: 419–439.

[29] Duffy SA, Morris RK, Rayner K. Lexical ambiguity and fixation times in reading. J Mem Lang. 1988; 27: 429–446.

[30] Schirmer A, Tang SL, Penney TB, Gunter TC, Chen HC. Brain responses to segmentally and tonally induced semantic violations in Cantonese. J Cogn Neurosci. 2005; 17: 1–12. 10.1162/0898929052880057 15701235

[31] Tsang YK, Wu Y, Ng HTY, Chen HC. Semantic activation of phonetic radicals in Chinese. Lan Cogn Neurosci. 2017; 32: 618–636.

[32] Barr DJ, Levy R, Scheepers C, Tily HJ. Random effects structure for confirmatory hypothesis testing: Keep it maximal. J Mem Lang. 2013; 68: 255–278. 10.1016/j.jml.2012.11.001 PMC3881361 24403724

[33] Taft M, Forster KI. Lexical storage and retrieval of prefixed words. J Verbal Learning Verbal Behav. 1975; 14: 638–647.

[34] Taft M. Interactive-activation as a framework for understanding morphological processing. Lang Cognitive Proc. 1994; 9: 271–294.

[35] Giraudo H, Grainger J. Priming complex words: Evidence for supralexical representation of morphology. Psychon Bull Rev. 2001; 8: 127–131. 10.3758/bf03196148 11340857

[36] Rastle K, Davis MH. Morphological decomposition based on the analysis of orthography. Lang Cognitive Proc. 2008; 23: 942–971.

[37] Feldman LB, O’Connor PA, del Prado Martín FM. Early morphological processing is morphosemantic and not simply morpho-orthographic: A violation of form-then-meaning accounts of word recognition. Psychon Bull Rev. 2009; 16: 684–691. 10.3758/PBR.16.4.684 19648453 PMC2883124

[38] Marelli M, Baroni M. Affixation in semantic space: Modeling morpheme meanings with compositional distributional semantics. Psychol Rev. 2015; 122: 485–515. 10.1037/a0039267 26120909

[39] Zwitserlood P. The role of semantic transparency in the processing and representation of Dutch compounds. Lang Cognitive Proc. 1994; 9: 341–368.

[40] Sandra D. On the representation and processing of compound words: Automatic access to constituent morphemes does not occur. Q J Exp Psychol. 1990; 42: 529–567.

[41] Beyersmann E, Coltheart M, Castles A. Parallel processing of whole words and morphemes in visual word recognition. Q J Exp Psychol. 2012; 65: 1798–1819. 10.1080/17470218.2012.672437 22540902

[42] Juhasz BJ. Sentence context modifies compound word recognition: Evidence from eye movements. J Cogn Psychol. 2012; 24: 855–870.

[43] Amenta S, Marelli M, Crepaldi D. The fruitless effort of growing a fruitless tree: Early morpho-orthographic and morpho-semantic effects in sentence reading. J Exp Psychol: Learn Mem Cogn. 2015; 41: 1587–1596. 25664370 10.1037/xlm0000104

[44] Chen HC, Cheung H, Tang SL, Wong YT. Effects of antecedent order and semantic context on Chinese pronoun resolution. Mem Cognit. 2000; 28: 427–438. 10.3758/bf03198558 10881560

